# Supplementary material for: Drivers and trends of global soil microbial carbon over two decades
Source: Nat Commun. 2022 Jul 20;13:4195. doi: 10.1038/s41467-022-31833-z (PMC9300697; doi:10.1038/s41467-022-31833-z)
Supplement: Supplementary file 1 — Supplementary Information [file 41467_2022_31833_MOESM1_ESM.pdf]

Supplementary Information for

Drivers and trends of global soil microbial carbon over two decades

Guillaume Patoine, Nico Eisenhauer, Simone Cesarz, Helen RP Phillips, Xiaofeng Xu, Lihua Zhang, Carlos A Guerra

This PDF file includes:
Supplementary Figures 1–8
Supplementary Tables 1–4

Supplementary Figures

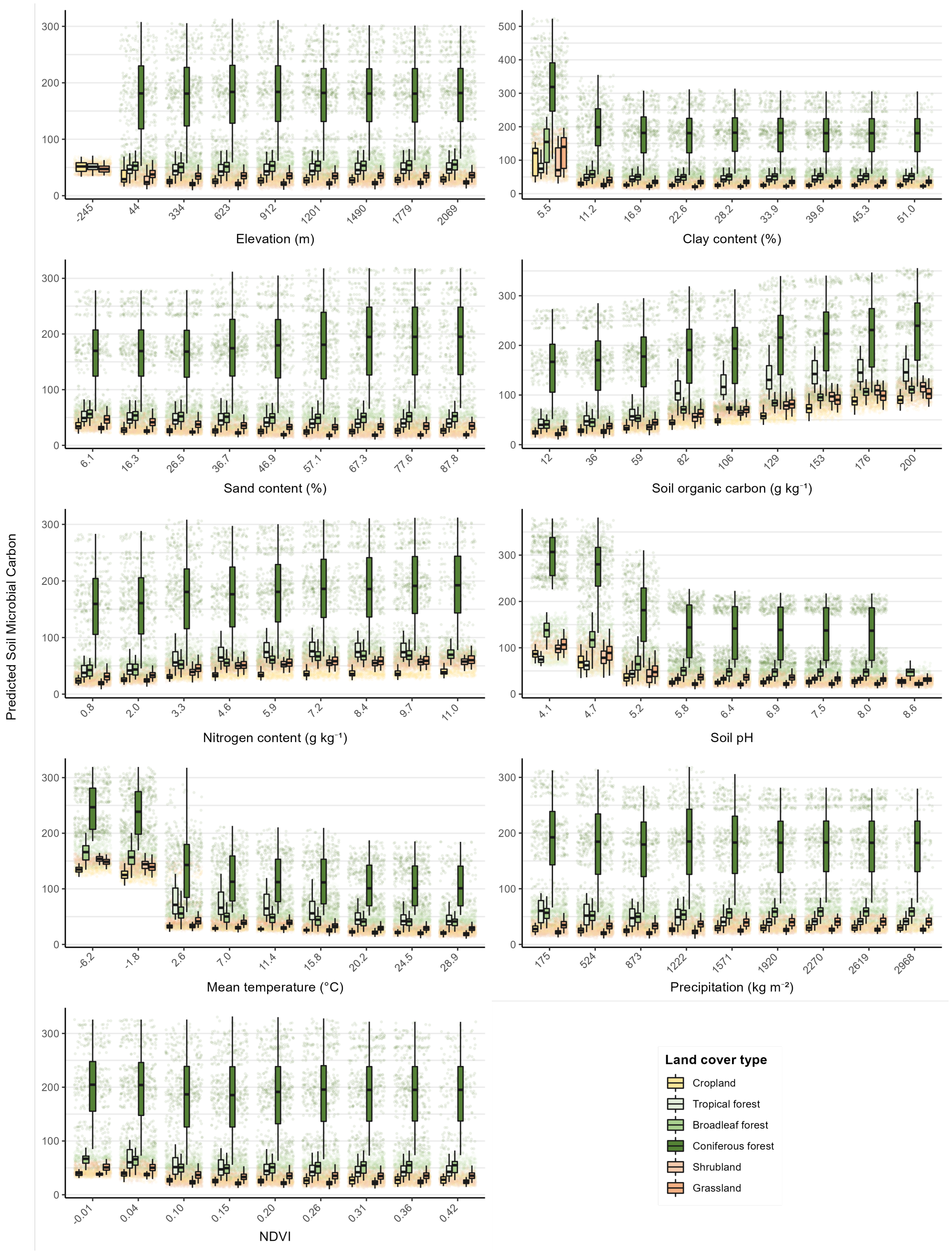

Supplementary Figure 1. Model predictions from generated data over the prediction range aggregated in bins, while using fixed values for the other variables. Values were not predicted beyond the range of location values, in which cases boxplots are missing. n = 230,400 prediction points generated, for 1536 partial prediction curves. Center line, median; box limits, upper and lower quartiles; whiskers, 1.5x interquartile range.

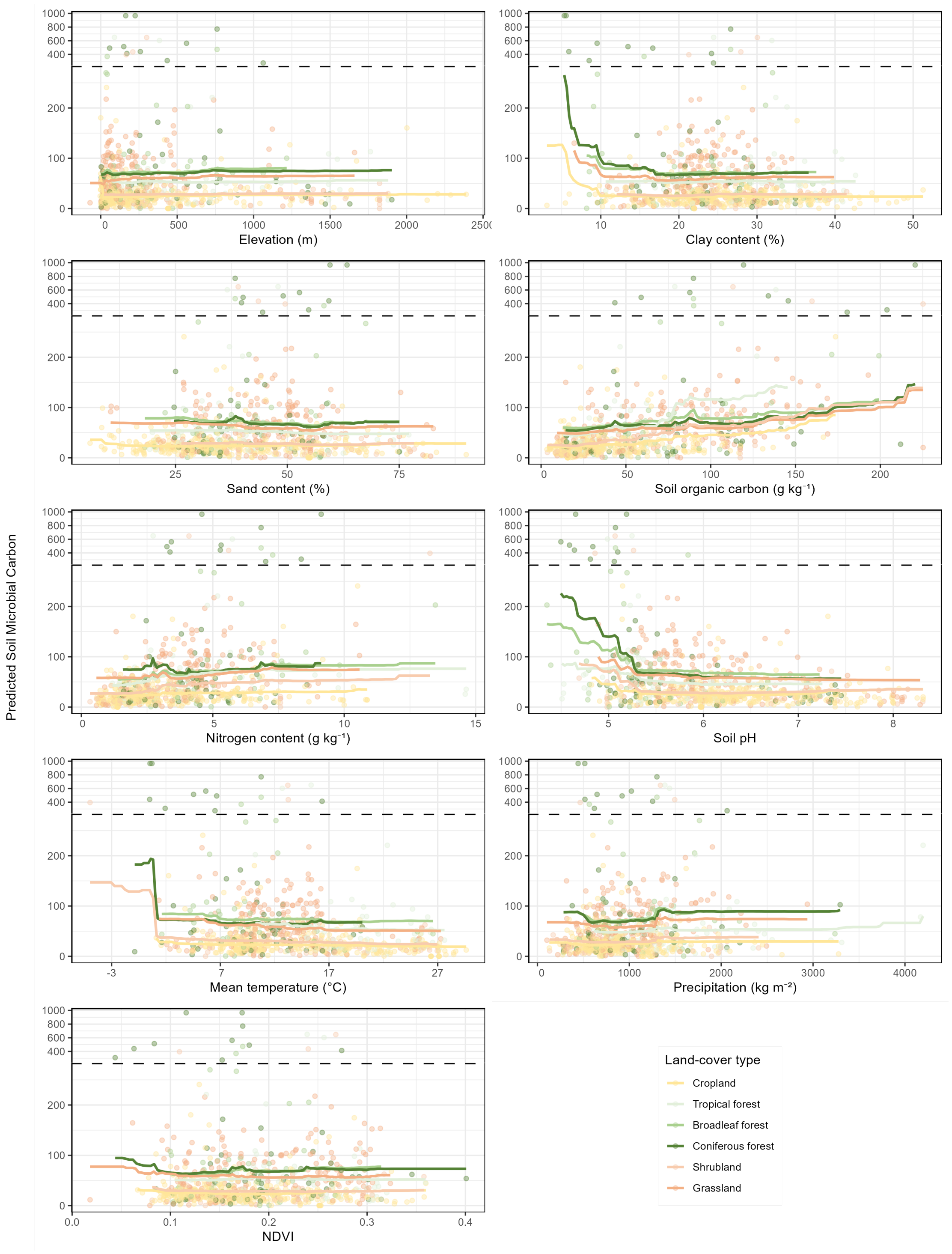

Supplementary Figure 2. Partial dependence plots showing the predicted relationship between microbial carbon and the nine variables, for each of the land-cover types. Variables were set to their median value to make predictions for each variable of interest. Data points are added. The y-axis changes scale at the dashed line, due to skewed microbial carbon values.

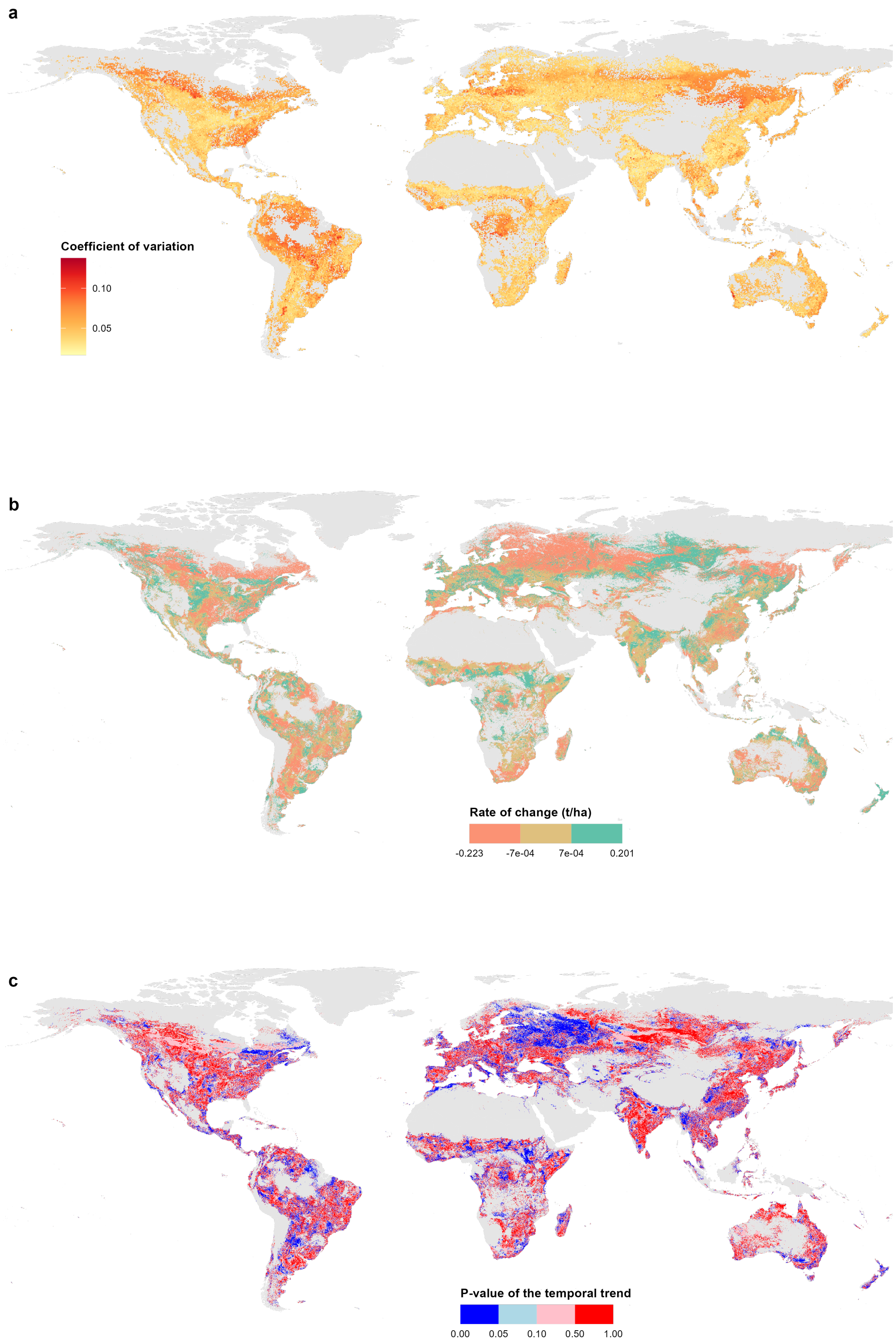

Supplementary Figure 3. a) Prediction coefficient of variation, i.e.SD divided by mean predicted value for 100 runs. b) Microbial carbon stock rate of change in tonnes/ha per year. c) P-value of microbial carbon rate of change, from two-sided linear regressions. Each location was modelled independently. No adjustments were made for multiple comparisons.

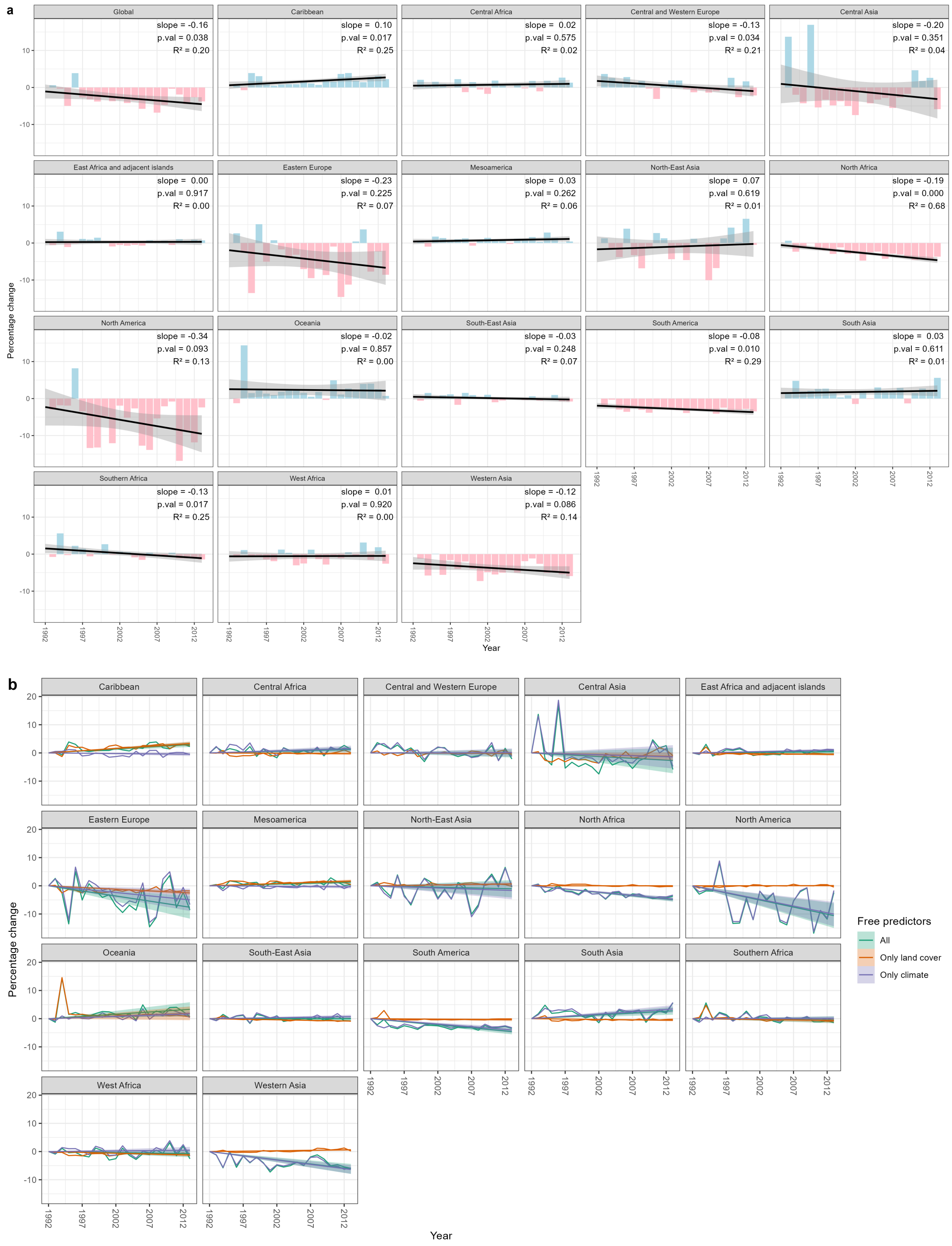

Supplementary Figure 4. a) Regional temporal microbial carbon stock dynamics in percentage change from 1992. Reported statistical results from two-sided linear regressions. b) Comparison of model predictions with either fixed climatic (temperature and precipitation) or land cover (land cover type and NDVI) variables. Grey areas represent 95% CI.

a. Slope annual temperature (range = -1.16 to 2.01)

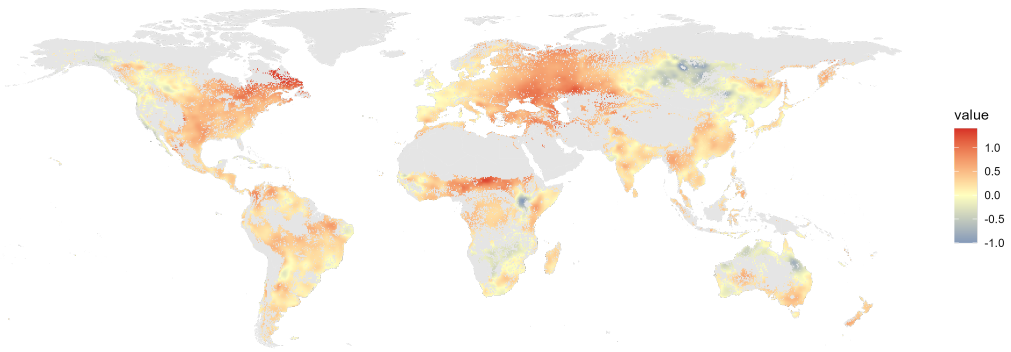

b. Slope annual precipitation (range = -366 to 124)

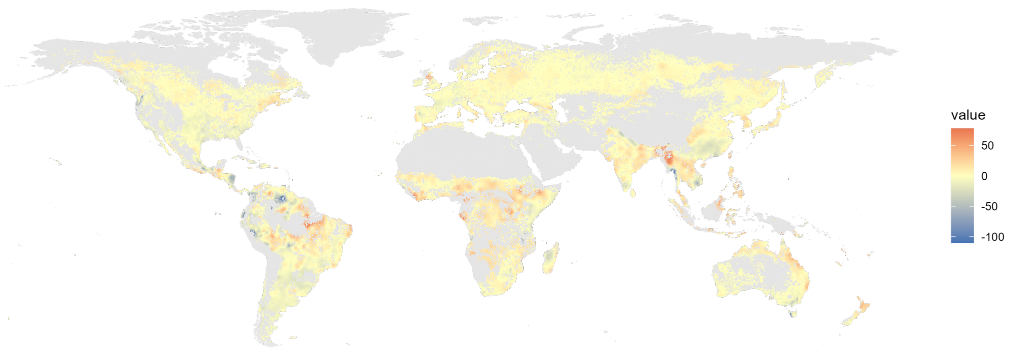

c. Slope annual NDVI (range = -0.0117 to 0.0138)

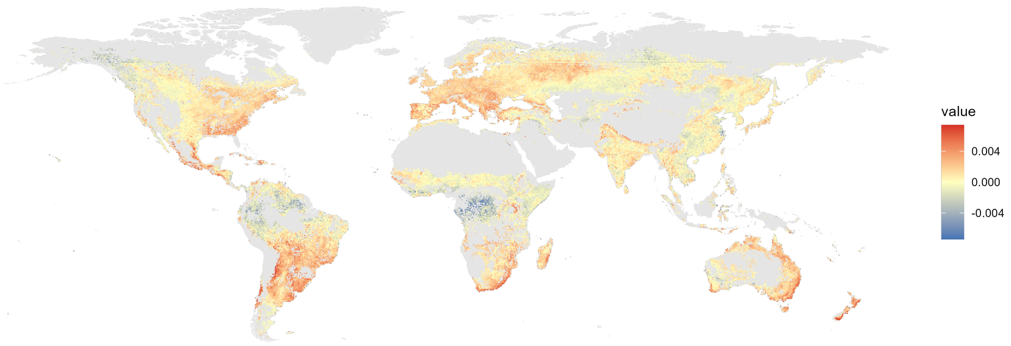

Supplementary Figure 5. a) Change in temperature (°C). b) Change in precipitation. c) Change in NDVI.

a

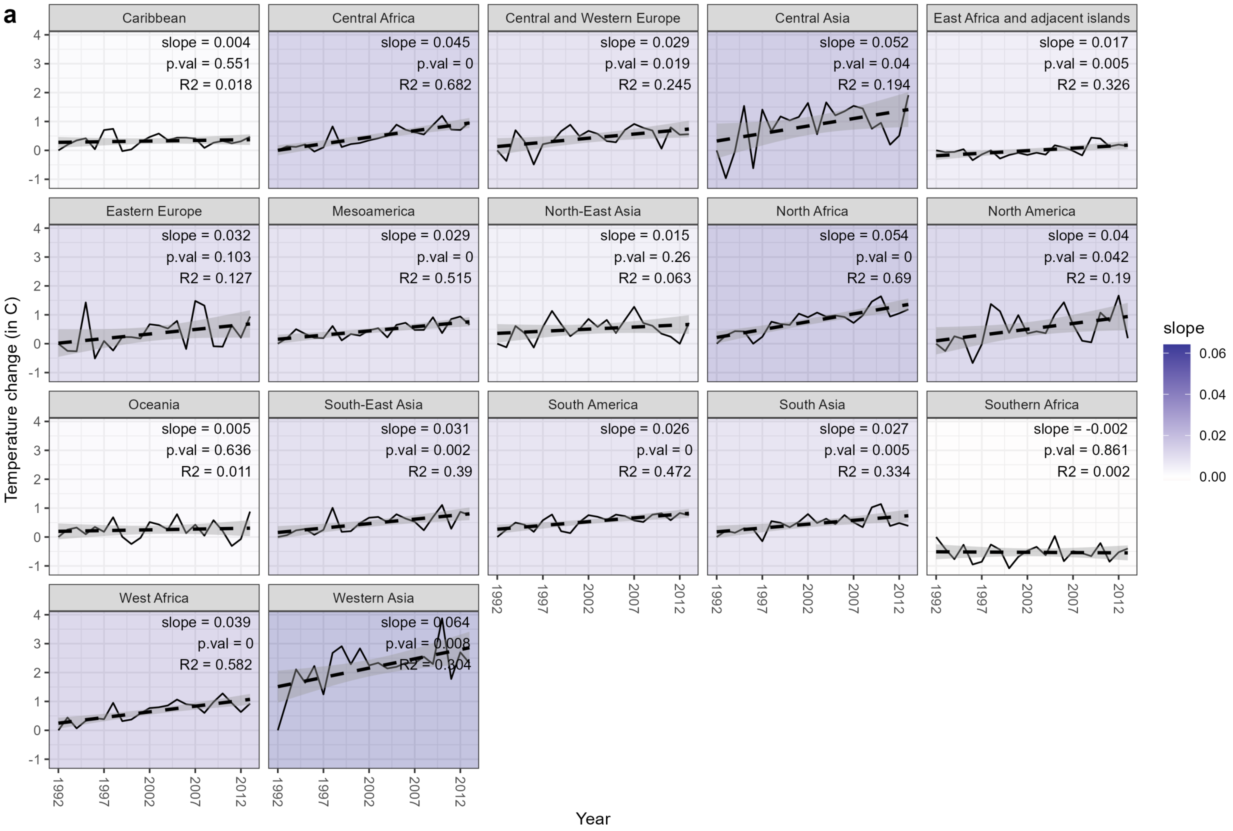
Caribbean
slope = 0.004
p.val = 0.551
R2 = 0.018
Central Africa
slope = 0.045
p.val = 0
R2 = 0.682
Central and Western Europe
slope = 0.029
p.val = 0.019
R2 = 0.245
Central Asia
slope = 0.052
p.val = 0.04
R2 = 0.194
East Africa and adjacent islands
slope = 0.017
p.val = 0.005
R2 = 0.326
Eastern Europe
slope = 0.032
p.val = 0.103
R2 = 0.127
Mesoamerica
slope = 0.029
p.val = 0
R2 = 0.515
North-East Asia
slope = 0.015
p.val = 0.26
R2 = 0.063
North Africa
slope = 0.054
p.val = 0
R2 = 0.69
North America
slope = 0.04
p.val = 0.042
R2 = 0.19
Oceania
slope = 0.005
p.val = 0.636
R2 = 0.011
South-East Asia
slope = 0.031
p.val = 0.002
R2 = 0.39
South America
slope = 0.026
p.val = 0
R2 = 0.472
South Asia
slope = 0.027
p.val = 0.005
R2 = 0.334
Southern Africa
slope = -0.002
p.val = 0.861
R2 = 0.002
West Africa
slope = 0.039
p.val = 0
R2 = 0.582
Western Asia
slope = 0.064
p.val = 0.008
R2 = 0.304
Temperature change (in C)
Year
slope
0.06
0.04
0.02
0.00

b

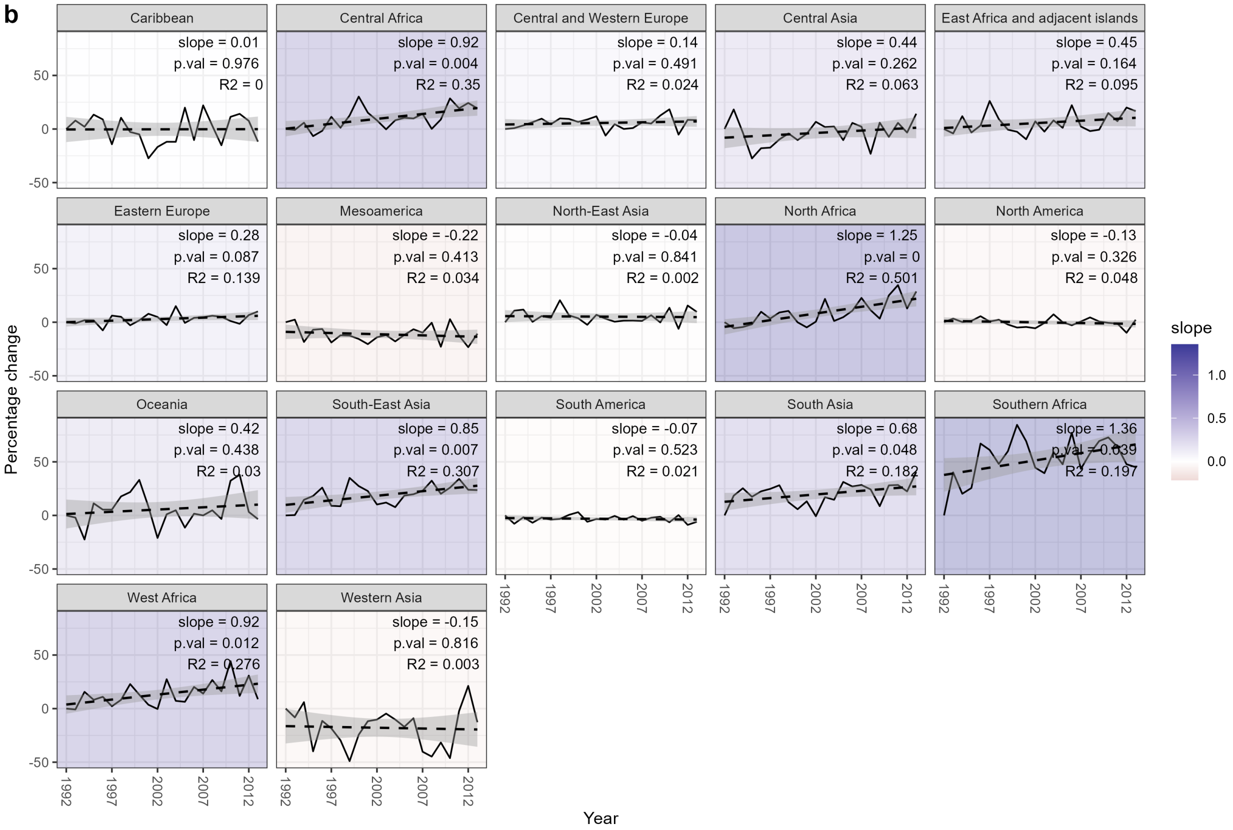
Caribbean
slope = 0.01
p.val = 0.976
R2 = 0
Central Africa
slope = 0.92
p.val = 0.004
R2 = 0.35
Central and Western Europe
slope = 0.14
p.val = 0.491
R2 = 0.024
Central Asia
slope = 0.44
p.val = 0.262
R2 = 0.063
East Africa and adjacent islands
slope = 0.45
p.val = 0.164
R2 = 0.095
Eastern Europe
slope = 0.28
p.val = 0.087
R2 = 0.139
Mesoamerica
slope = -0.22
p.val = 0.413
R2 = 0.034
North-East Asia
slope = -0.04
p.val = 0.841
R2 = 0.002
North Africa
slope = 1.25
p.val = 0
R2 = 0.501
North America
slope = -0.13
p.val = 0.326
R2 = 0.048
Oceania
slope = 0.42
p.val = 0.438
R2 = 0.03
South-East Asia
slope = 0.85
p.val = 0.007
R2 = 0.307
South America
slope = -0.07
p.val = 0.523
R2 = 0.021
South Asia
slope = 0.68
p.val = 0.048
R2 = 0.182
Southern Africa
slope = 1.36
p.val = 0.039
R2 = 0.197
West Africa
slope = 0.92
p.val = 0.012
R2 = 0.276
Western Asia
slope = -0.15
p.val = 0.816
R2 = 0.003
Percentage change
Year
slope
1.0
0.5
0.0

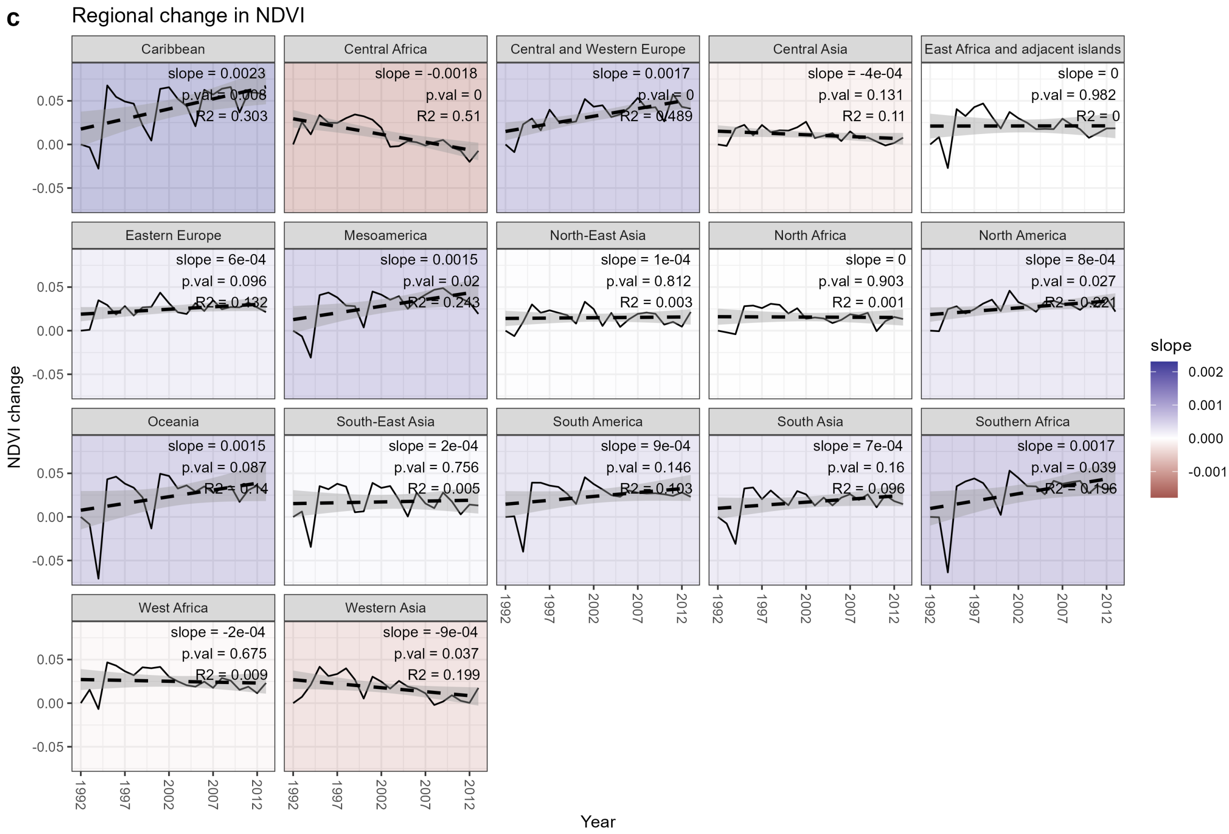

Supplementary Figure 6. Regional changes in a) temperature (°C), b) relative precipitation, and c) NDVI. Grey areas represent 95% CI. Reported statistical results from two-sided linear regressions.

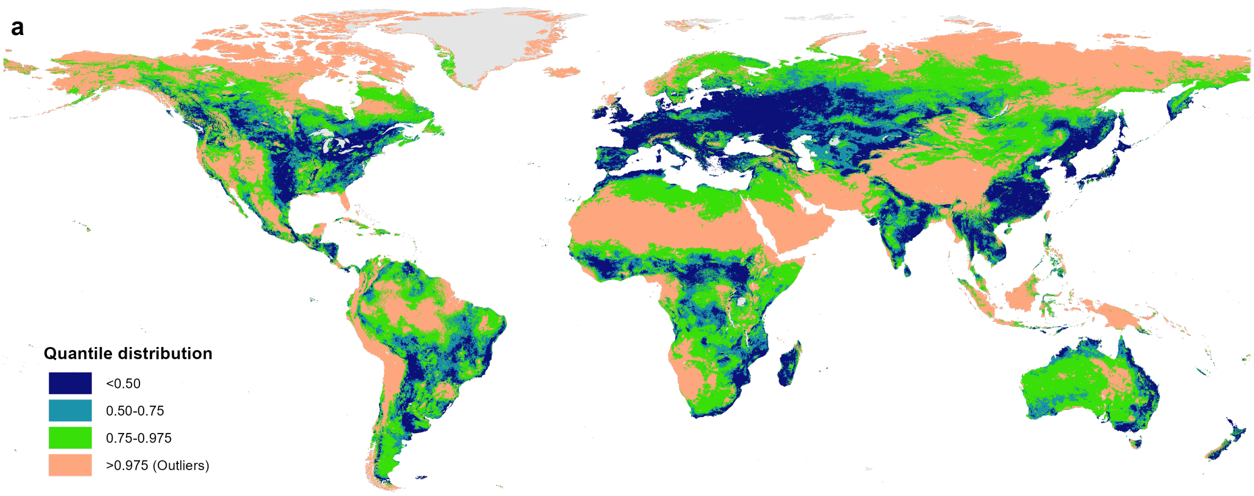

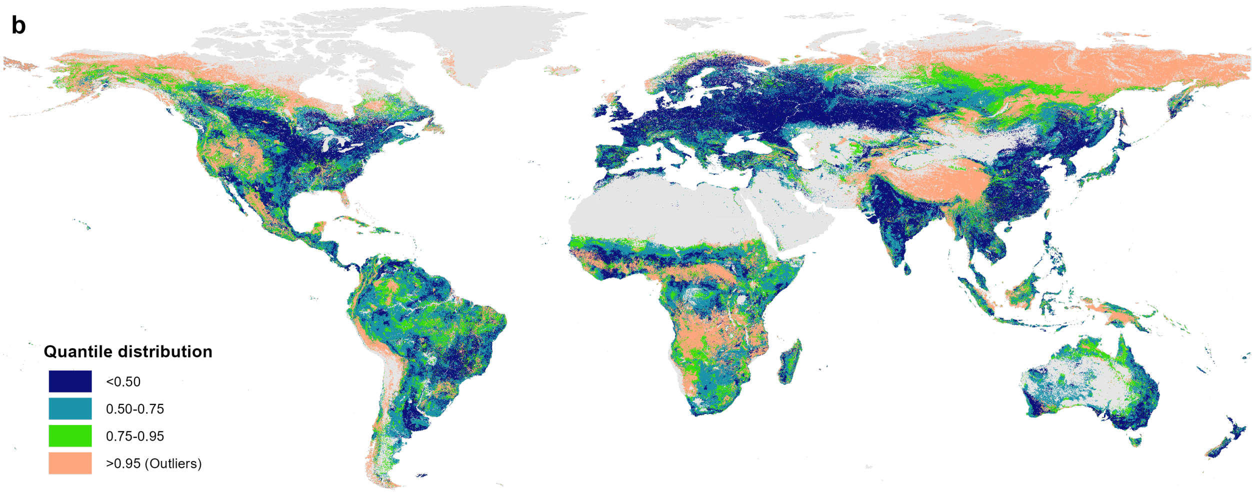

Supplementary Figure 7. a) Mahalanobis quantile distribution. b) Area of applicability (AOA) quantile distribution.

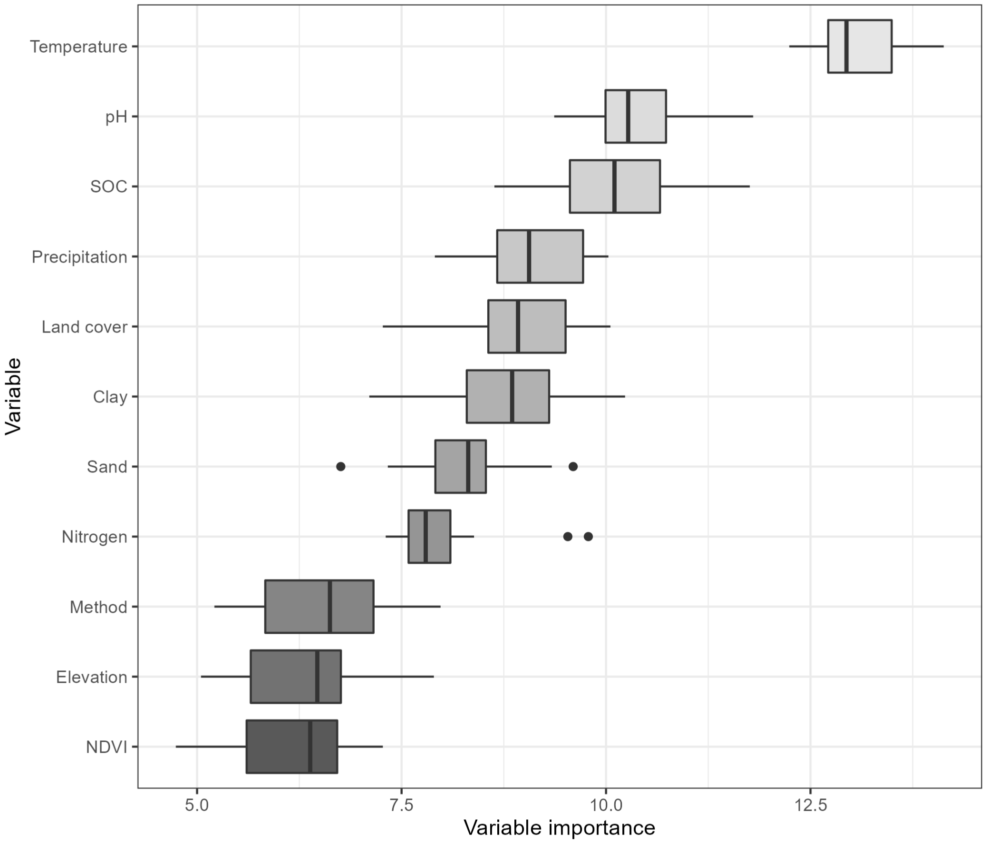

Supplementary Figure 8. Distribution of the variable importance for 20 random forest models including the measurement method as variable. As the variable importance remains very low, we can conclude that the sampling method does not have an important influence on the reported microbial carbon values between studies. Center line, median; box limits, upper and lower quartiles; whiskers, 1.5x interquartile range; points, outliers.

Supplementary Tables

Supplementary Table 1. Regional changes in soil microbial carbon stocks per IPBES regions from the models using either all free predictors, or fixed values for land cover (land cover type and NDVI) or climatic (temperature and precipitation) variables. Change per year and total between 1992 and 2013 are given in absolute and relative values, with the 95% confidence interval (CI) for relative yearly change and R^2 value. Cases where the rate of change is higher than the 95% CI are shown in bold.

Free predictors: All

	Relative (% per year)			Absolute (tonnes)		
IPBES region	Rate of change	95% CI	Total over 22 y	Rate of change	Total over 22 y	R^2
Global	**−0.163**	**0.144**	−3.43	−7,085,952	−148,804,991	0.198
Central Africa	0.024	0.081	0.49	27,730	582,328	0.016
East Africa and adjacent islands	0.004	0.066	0.07	3,476	73,004	0.001
North Africa	**−0.195**	**0.059**	−4.09	−57,979	−1,217,569	0.676
Southern Africa	**−0.125**	**0.095**	−2.64	−116,884	−2,454,563	0.252
West Africa	0.006	0.109	0.12	3,778	79,331	0.001
Caribbean	**0.100**	**0.076**	2.10	5,533	116,197	0.252
Mesoamerica	0.033	0.056	0.69	18,820	395,212	0.062
North America	−0.345	0.383	−7.24	−2,975,581	−62,487,192	0.135
South America	**−0.083**	**0.057**	−1.74	−464,914	−9,763,189	0.287
North-East Asia	0.069	0.267	1.44	213,943	4,492,803	0.013
Oceania	−0.019	0.206	−0.40	−30,051	−631,063	0.002
South-East Asia	−0.034	0.057	−0.72	−34,756	−729,878	0.066
South Asia	0.030	0.112	0.62	33,912	712,156	0.013
Western Asia	−0.121	0.131	−2.53	−7,511	−157,722	0.140
Central and Western Europe	**−0.130**	**0.112**	−2.74	−481,356	−10,108,477	0.205
Central Asia	−0.195	0.401	−4.10	−198,014	−4,158,292	0.044
Eastern Europe	−0.226	0.353	−4.74	−2,899,122	−60,881,564	0.073

Supplementary Table 1. (continued)

Free predictors: Only land cover

	Relative (% per year)			Absolute (tonnes)		
IPBES region	Rate of change	95% CI	Total over 22 y	Rate of change	Total over 22 y	R^2
Global	−0.010	0.022	−0.22	−465,785	−9,781,494	0.043
Central Africa	0.032	0.038	0.67	37,360	784,554	0.121
East Africa and adjacent islands	−0.025	0.037	−0.53	−24,860	−522,062	0.085
North Africa	**−0.026**	**0.019**	−0.55	−7,994	−167,871	0.271
Southern Africa	**−0.099**	**0.064**	−2.09	−92,286	−1,938,000	0.319
West Africa	−0.003	0.028	−0.06	−1,753	−36,818	0.002
Caribbean	**0.121**	**0.057**	2.55	6,713	140,982	0.461
Mesoamerica	0.016	0.036	0.34	9,271	194,690	0.038
North America	**0.026**	**0.020**	0.54	235,329	4,941,915	0.241
South America	−0.033	0.046	−0.69	−188,268	−3,953,623	0.089
North-East Asia	0.003	0.027	0.07	10,195	214,102	0.003
Oceania	−0.112	0.190	−2.35	−174,081	−3,655,702	0.062
South-East Asia	**−0.052**	**0.018**	−1.10	−52,603	−1,104,672	0.608
South Asia	−0.013	0.028	−0.27	−14,348	−301,304	0.039
Western Asia	**0.047**	**0.024**	0.99	3,072	64,509	0.418
Central and Western Europe	0.009	0.047	0.20	34,521	724,934	0.008
Central Asia	**0.111**	**0.092**	2.33	112,169	2,355,559	0.217
Eastern Europe	−0.027	0.051	−0.57	−358,414	−7,526,697	0.052

Supplementary Table 1. (continued)

Free predictors: Only climate

	Relative (% per year)			Absolute (tonnes)		
IPBES region	Rate of change	95% CI	Total over 22 y	Rate of change	Total over 22 y	R^2
Global	**−0.146**	**0.141**	−3.06	−6,430,941	−135,049,757	0.169
Central Africa	−0.015	0.083	−0.32	−18,000	−378,004	0.006
East Africa and adjacent islands	0.032	0.047	0.68	31,964	671,254	0.082
North Africa	**−0.164**	**0.058**	−3.44	−49,197	−1,033,132	0.603
Southern Africa	−0.024	0.062	−0.51	−22,625	−475,123	0.028
West Africa	0.013	0.102	0.26	8,506	178,618	0.003
Caribbean	−0.025	0.060	−0.52	−1,373	−28,826	0.032
Mesoamerica	0.019	0.051	0.39	10,668	224,037	0.026
North America	−0.350	0.380	−7.36	−3,076,870	−64,614,269	0.141
South America	−0.044	0.048	−0.92	−249,574	−5,241,046	0.139
North-East Asia	0.068	0.268	1.43	214,227	4,498,770	0.012
Oceania	**0.087**	**0.070**	1.83	138,258	2,903,420	0.228
South-East Asia	0.015	0.055	0.32	15,737	330,475	0.015
South Asia	0.046	0.101	0.96	53,158	1,116,310	0.038
Western Asia	**−0.150**	**0.114**	−3.14	−9,542	−200,385	0.250
Central and Western Europe	**−0.129**	**0.111**	−2.71	−484,986	−10,184,704	0.207
Central Asia	−0.297	0.348	−6.23	−308,092	−6,469,930	0.122
Eastern Europe	−0.195	0.351	−4.09	−2,573,632	−54,046,267	0.056

Supplementary Table 2. Number of locations (pixels) and area included in predictions for each IPBES sub-region, with total stock of microbial carbon (mean value for 1992-2013).

	Pixels			Area (km^2)			Microbial carbon
Region	total	predicted	%	total	predicted	%	stock (t)
Caribbean	7,026	3,640	51.8	202,984	105,651	52.0	5,525,183
Central Africa	165,099	76,199	46.2	5,014,912	2,329,589	46.5	117,789,621
Central and Western Europe	283,211	230,305	81.3	5,414,839	4,588,599	84.7	369,248,503
Central Asia	178,207	90,686	50.9	3,807,241	1,866,019	49.0	101,420,270
East Africa and adjacent islands	153,813	96,871	63.0	4,662,199	2,936,987	63.0	98,376,058
Eastern Europe	1,102,870	511,331	46.4	16,292,736	8,793,579	54.0	1,284,732,309
Mesoamerica	84,535	47,598	56.3	2,401,290	1,359,993	56.6	57,037,690
North-East Asia	464,160	213,261	45.9	11,135,132	5,166,004	46.4	311,609,366
North Africa	308,765	32,435	10.5	8,596,638	908,873	10.6	29,768,320
North America	1,004,265	432,558	43.1	17,227,758	9,108,348	52.9	863,396,351
Oceania	301,014	157,174	52.2	8,299,006	4,271,699	51.5	156,743,166
South-East Asia	138,588	71,608	51.7	4,154,586	2,124,617	51.1	101,226,217
South America	584,372	370,751	63.4	16,793,472	10,683,050	63.6	561,526,577
South Asia	238,602	119,380	50.0	6,475,524	3,326,630	51.4	114,432,966
Southern Africa	201,587	96,516	47.9	5,798,925	2,741,259	47.3	93,137,630
West Africa	165,120	71,671	43.4	4,922,324	2,163,804	44.0	66,578,411
Western Asia	125,515	7,035	5.6	3,486,501	178,883	5.1	6,230,514

Supplementary Table 3. Land cover reclassification table, based on the ESA CCI gridded layer. Land cover classes defined as *NA* (not assigned) were not used for this analysis.

Land cover class	ESA code	Description
Cropland	10	Cropland, rainfed
Cropland	11	Herbaceous cover
Cropland	12	Tree or shrub cover
Cropland	20	Cropland, irrigated or post-flooding
Cropland	30	Mosaic cropland (>50%) / natural vegetation (tree, shrub, herbaceous cover) (<50%)
Cropland	40	Mosaic natural vegetation (tree, shrub, herbaceous cover) (>50%) / cropland (<50%)
Tropical forest	50	Tree cover, broadleaved, evergreen, closed to open (>15%)
Broadleaf forest	60	Tree cover, broadleaved, deciduous, closed to open (>15%)
Broadleaf forest	61	Tree cover, broadleaved, deciduous, closed (>40%)
Broadleaf forest	62	Tree cover, broadleaved, deciduous, open (15-40%)
Coniferous forest	70	Tree cover, needleleaved, evergreen, closed to open (>15%)
Coniferous forest	71	Tree cover, needleleaved, evergreen, closed (>40%)
Coniferous forest	72	Tree cover, needleleaved, evergreen, open (15-40%)
Coniferous forest	80	Tree cover, needleleaved, deciduous, closed to open (>15%)
Coniferous forest	81	Tree cover, needleleaved, deciduous, closed (>40%)
Coniferous forest	82	Tree cover, needleleaved, deciduous, open (15-40%)
Coniferous forest	90	Tree cover, mixed leaf type (broadleaved and needleleaved)
Shrubland	100	Mosaic tree and shrub (>50%) / herbaceous cover (<50%)
Grassland	110	Mosaic herbaceous cover (>50%) / tree and shrub (<50%)
Shrubland	120	Shrubland
Shrubland	121	Evergreen shrubland
Shrubland	122	Deciduous shrubland
Grassland	130	Grassland
NA	140	Lichens and mosses
NA	150	Sparse vegetation (tree, shrub, herbaceous cover) (<15%)
NA	160	Tree cover, flooded, fresh or brakish water
NA	170	Tree cover, flooded, saline water
NA	180	Shrub or herbaceous cover, flooded, fresh/saline/brakish water
NA	190	Urban areas
NA	200	Bare areas
NA	210	Water bodies
NA	220	Permanent snow and ice

Supplementary Table 4. List of environmental variables used

Variable	Category	Source	Description	Reference
Elevation	geography	WorldClim	Elevation above sea level	Fick and Hijmans, 2017
Soil organic carbon	soil properties	SoilGrids	Three depths layers, mean values	Hengl et al., 2017
Soil total nitrogen	soil properties	SoilGrids	Three depths layers, mean values	Hengl et al., 2017
Soil pH	soil properties	SoilGrids	Three depths layers, mean values	Hengl et al., 2017
Soil sand portion	soil properties	SoilGrids	Three depths layers, mean values	Hengl et al., 2017
Soil clay portion	soil properties	SoilGrids	Three depths layers, mean values	Hengl et al., 2017
Yearly mean temperature	climate	CHELSA	1979-2013, monthly values averaged	Karger et al., 2018
Yearly total precipitation	climate	CHELSA	1979-2013, monthly values summed	Karger et al., 2018
Land cover	land cover	ESA	1992-2015, reclassified	ESA, 2017
Normalized Difference Vegetation Index (NDVI)	vegetation	NOAA	1981-2016, daily values averaged	Vermote et al., 2018
