# Supplementary material for: Drivers and trends of global soil microbial carbon over two decades
Source: Nat Commun. 2022 Jul 20;13:4195. doi: 10.1038/s41467-022-31833-z (PMC9300697; doi:10.1038/s41467-022-31833-z)
Supplement: Supplementary file 3 — Description of Additional Supplementary Files [file 41467_2022_31833_MOESM3_ESM.pdf]

Description of Additional Supplementary Files

File Name: Supplementary Movie 1
Description: Yearly microbial carbon stock predictions from 1992 to 2013. While overall spatial patterns remain, the large yearly variations hide important temporal trends.
