# Supplementary figures and images for: Drivers and trends of global soil microbial carbon over two decades
Source: Nat Commun. 2022 Jul 20;13:4195. doi: 10.1038/s41467-022-31833-z (PMC9300697; doi:10.1038/s41467-022-31833-z)

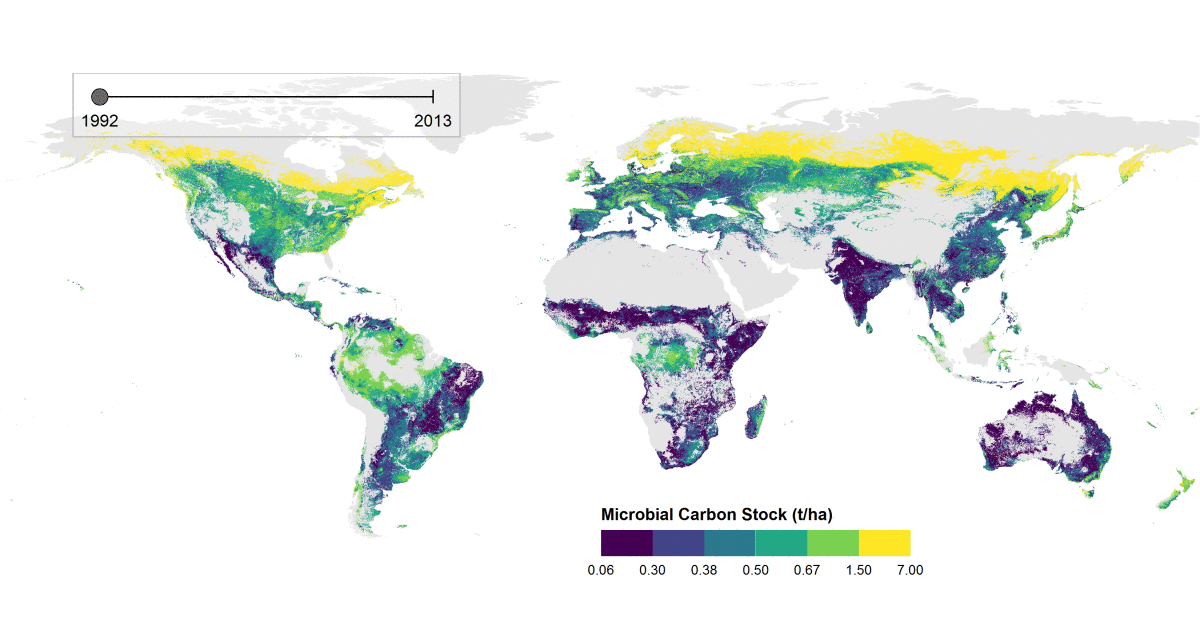

Supplement: Supplementary file 4 — Supplementary Movie 1 [file 41467_2022_31833_MOESM4_ESM.gif]
