# Supplementary material for: Drivers and trends of global soil microbial carbon over two decades
Source: Nat Commun. 2022 Jul 20;13:4195. doi: 10.1038/s41467-022-31833-z (PMC9300697; doi:10.1038/s41467-022-31833-z)
Supplement: Supplementary file 5 — Reporting Summary [file 41467_2022_31833_MOESM5_ESM.pdf]

nature portfolio

Corresponding author(s): Guillaume Patoine

Last updated by author(s): Jun 17, 2022

Reporting Summary

Nature Portfolio wishes to improve the reproducibility of the work that we publish. This form provides structure for consistency and transparency in reporting. For further information on Nature Portfolio policies, see our Editorial Policies and the Editorial Policy Checklist.

Statistics

For all statistical analyses, confirm that the following items are present in the figure legend, table legend, main text, or Methods section.

n/a	Confirmed	
☐	☒	The exact sample size (*n*) for each experimental group/condition, given as a discrete number and unit of measurement
☐	☒	A statement on whether measurements were taken from distinct samples or whether the same sample was measured repeatedly
☐	☒	The statistical test(s) used AND whether they are one- or two-sided<br>*Only common tests should be described solely by name; describe more complex techniques in the Methods section.*
☐	☒	A description of all covariates tested
☐	☒	A description of any assumptions or corrections, such as tests of normality and adjustment for multiple comparisons
☐	☒	A full description of the statistical parameters including central tendency (e.g. means) or other basic estimates (e.g. regression coefficient) AND variation (e.g. standard deviation) or associated estimates of uncertainty (e.g. confidence intervals)
☐	☒	For null hypothesis testing, the test statistic (e.g. *F*, *t*, *r*) with confidence intervals, effect sizes, degrees of freedom and *P* value noted<br>*Give P values as exact values whenever suitable.*
☒	☐	For Bayesian analysis, information on the choice of priors and Markov chain Monte Carlo settings
☐	☒	For hierarchical and complex designs, identification of the appropriate level for tests and full reporting of outcomes
☐	☒	Estimates of effect sizes (e.g. Cohen's *d*, Pearson's *r*), indicating how they were calculated

Our web collection on statistics for biologists contains articles on many of the points above.

Software and code

Policy information about availability of computer code

Data collection	We did not use code to collect the data used in this study.
Data analysis	Data analysis was performed with R software (version 3.6.3), and the packages "tidyverse" (version 1.3.1), "raster" (version 3.5.15), "caret" (version 6.0.86), "randomForest" (version 4.7.1), and "CAST" (version 0.6.0). The code can be accessed at https://zenodo.org/record/6645922.

For manuscripts utilizing custom algorithms or software that are central to the research but not yet described in published literature, software must be made available to editors and reviewers. We strongly encourage code deposition in a community repository (e.g. GitHub). See the Nature Portfolio guidelines for submitting code & software for further information.

Data

Policy information about availability of data

All manuscripts must include a data availability statement. This statement should provide the following information, where applicable:

- Accession codes, unique identifiers, or web links for publicly available datasets
- A description of any restrictions on data availability
- For clinical datasets or third party data, please ensure that the statement adheres to our policy

The microbial carbon dataset was deposited in the public data repository Zenodo and can be accessed at https://zenodo.org/record/6645922. The publicly available global gridded datasets used as covariates in this study are: WorldClim (Fick and Hijmans, 2017), SoilGrids (Hengl et al., 2017), CHELSA (Karger et al., 2018), ESA CCI Land Cover (ESA, 2017), and NOAA NDVI (Vermote et al., 2018). See Supplementary Table 4 for additional information

Field-specific reporting

Please select the one below that is the best fit for your research. If you are not sure, read the appropriate sections before making your selection.

☐ Life sciences ☐ Behavioural & social sciences ☒ Ecological, evolutionary & environmental sciences

For a reference copy of the document with all sections, see nature.com/documents/nr-reporting-summary-flat.pdf

Ecological, evolutionary & environmental sciences study design

All studies must disclose on these points even when the disclosure is negative.

Study description	This study assesses the temporal trends in soil microbial biomass carbon and identifies the main drivers of change globally and regionally. We used a global soil microbial biomass carbon dataset with 762 independent samples compiled from the literature, random forest modeling, and global environmental layers to predict spatial-temporal dynamics of soil microbial biomass carbon from 1992 to 2013.
Research sample	The study uses data on soil microbial carbon measurements compiled from peer-reviewed articles. The original dataset is available at https://daac.ornl.gov/cgi-bin/dsviewer.pl?ds_id=1264
Sampling strategy	We used an existing dataset compiled from literature review. We aggregated datapoints by location, study, year, and land-cover type, for a final 762 independent entries that were used for modelling. We tested the effect of sample size on the model fit, which increased with sample size and started to plateau when using the full dataset. We performed an environmental coverage analysis to evaluated which areas can be predicted with high confidence with the dataset that we used.
Data collection	Soil microbial carbon data was performed by literature review and data extraction from published papers (see full method documented in Xu et al., 2013). We extracted all environmental variable data from global layers freely available (see Supplementary Table 4 for references).
Timing and spatial scale	We did not perform sampling and direct measurement for this experiment, as we used a precompiled soil microbial carbon dataset (Xu et al., 2013). The dataset incorporated studies which each sampled at one or multiple locations, and at one or multiple dates. Taken together, the sampling dates ranged from 1977 to 2016, and the spatial extend of the compiled dataset is global. See Figure 1 for sampling locations.
Data exclusions	From the available dataset, we selected entries with soil microbial carbon measurements sampled at a mean depth above 30 cm, in order to represent the upper soil part and match the global layers used. Sites from wetlands and bare areas (including both cold and warm deserts) were removed, as too few entries were available for proper statistical analysis and predictions (20 and 31 entries, respectively). Selected entries from the same study were then aggregated (averaged) at each sampling location and year.
Reproducibility	We performed a modeling variability assessment using a set of 100 models and compared the variable importance and predictions. The results of this assessment is presented in the text and described in Figure 3a and Supp. Figure 5a.
Randomization	We did not separate samples into treatments. We used random forest modeling, which selects subset of samples for training and evaluation at each cross-validation run.
Blinding	Blinding was not relevant for our study as we did not perform a field or laboratory experiment. Personal judgement or interpretation was not required. Our results were derived from quantitative computer pipelines with standardized procedures for all data points.

Did the study involve field work? ☐ Yes ☒ No

Reporting for specific materials, systems and methods

We require information from authors about some types of materials, experimental systems and methods used in many studies. Here, indicate whether each material, system or method listed is relevant to your study. If you are not sure if a list item applies to your research, read the appropriate section before selecting a response.

Materials & experimental systems

n/a	Involved in the study	
☒	☐	Antibodies
☒	☐	Eukaryotic cell lines
☒	☐	Palaeontology and archaeology
☒	☐	Animals and other organisms
☒	☐	Human research participants
☒	☐	Clinical data
☒	☐	Dual use research of concern

Methods

n/a	Involved in the study	
☒	☐	ChIP-seq
☒	☐	Flow cytometry
☒	☐	MRI-based neuroimaging
